# A Novel Pathogenic Variant in the *KRT3* Gene in a Family with Meesmann Corneal Dystrophy

**DOI:** 10.3390/jcm14030851

**Published:** 2025-01-28

**Authors:** Alix De Faria, Víctor Charoenrook, Raquel Larena, Álvaro Ferragut-Alegre, Rebeca Valero, Gemma Julio, Rafael I. Barraquer

**Affiliations:** 1 Centro de Oftalmología Barraquer, 08021 Barcelona, Spain; defariaalix@gmail.com (A.D.F.); charoenrook@barraquer.com (V.C.); raquel.larena@barraquer.com (R.L.); alvaroferragut@gmail.com (Á.F.-A.); prof.rafael@barraquer.com (R.I.B.); 2 Institut Universitari Barraquer, Universitat Autònoma de Barcelona, 08021 Barcelona, Spain; 3 DBGen Ocular Genomics, 08028 Barcelona, Spain; rvalero@dbgen.com; 4 School of Medicine, Universitat Internacional de Catalunya, 08193 Barcelona, Spain

**Keywords:** Meesmann corneal dystrophy, KRT3, KRT12, in vivo confocal microscopy, anterior segment optical coherence tomography, corneal epithelium

## Abstract

**Background/Objectives:** to report a novel KRT3 Meesmann corneal dystrophy (MECD) mutation and its clinical findings in a Spanish family, thus completing the international database. Case series study. **Methods:** Two generations of three family members were studied. The clinical ophthalmologic evaluation was made including best-corrected visual acuity (BCVA), biomicroscopy with and without fluorescein, fundoscopy, Schirmer test I, non-invasive break-up time (NiBUT), and esthesiometry. In vivo confocal microscopy (IVCM), anterior segment optical coherence tomography (AS-OCT) with an epithelial map, and genetic analysis were also performed. **Results:** A novel heterozygous mutation in the KRT3 gene c.1527G>T (p. Glu509Asp) was identified. Biomicroscopy revealed bilateral multiple corneal intraepithelial cysts. IVCM showed numerous and relatively small microcysts (12–32 µm), hyperreflective materials, subepithelial nerve and Bowman’s layer alterations. AS-OCT scan revealed diffuse hyperreflectivity and the epithelial map displayed thickening of the corneal epithelium in the interpalpebral zone (proband: 52–68 µm and father’s proband: 55–71 µm) with a slightly thinned cornea. **Conclusions:** We identified a new mutation in the KRT3 gene–c.1527G>T (p. Glu509Asp) in a Spanish family with MECD. A comprehensive characterization of the clinical signs, using different techniques, especially an epithelial map, could be useful to diagnose and monitor epithelial changes by quantitative measures. Epithelial map changes provide better understanding of MECD differential epithelial behavior and its progression changes. Larger studies will be necessary to better understand these specific patterns and clinically evaluate new therapies.

## 1. Introduction

Meesmann corneal dystrophy (MECD), also known as juvenile hereditary epithelial dystrophy (MECD), is a rare epithelial corneal disease that has an autosomal dominant inheritance [1,2] and incomplete penetrance [3,4,5]. Signs and symptoms were first reported by Pameijer in 1935 [1] and completely described by Meesmann and Wilke in 1939 [2]. Ever since, cases have been described in Denmark [6], Germany [7], Japan [8,9], USA [10,11], China [12], Vietnam [13], Poland [14], and Spain [15].

Irvine and coworkers were the first to discover a genetic abnormality that causes MECD [16]. Initial genetic analyses revealed that mutations in the cornea-specific keratin 3 (KRT3) and keratin 12 (KRT12) genes are responsible for MECD [16,17]. To our knowledge, six KRT3 mutations and twenty-five KRT12 mutations have been associated with MECD [7,8,9,10,11,13,14,16,17,18,19,20,21,22,23,24,25,26,27,28] until now (Table 1).

MECD is diagnosed by biomicroscopy and characterized by bilateral microcysts and fine punctate opacities in the corneal epithelium [8,9,30] with onset as early as birth [13]. Although MECD may be asymptomatic, affected individuals usually experience ocular symptoms that include contact lens intolerance, foreign body sensation, tearing, recurrent corneal erosions, glare, mild visual acuity impairment, and photophobia, sometimes being misdiagnosed with dry eye syndrome [8,9,13,30]. Usually palliative treatment includes lubricants, cycloplegia, and therapeutic contact lenses. In severe cases, epithelial debridement [31] and phototherapeutic keratectomy [32] are the mainstay for treating corneal erosions in this dystrophy. Recently, a new therapy based on Immunosafe Plasma Rich in Growth Factors eyedrops (is-ePRGF) associated with an improvement on the third day was reported in a case report [33].

Some gene-based therapies have been used in MECD to reduce mutant K12 expression and aggregate formation, such as allele-specific small interfering RNA (iRNA), proved in a human MECD cell line with promising results [22,34,35]. In the same way, CRISPR/Cas9 were shown to be effective in inhibiting mutant K12 expression in vivo [36], but the efficacy and safety of these emerging therapies for clinical use are yet to be investigated [37]. New gene-based strategies have also been reported for other corneal dystrophies [5,37,38,39]. Therefore, novel mutation identification is necessary not only to avoid misdiagnosis but for implementing future gene-based strategies of treatment.

The implementation of new non-invasive corneal diagnostic imaging technologies such as laser scanning in vivo confocal microscopy (IVCM) and anterior segment optical coherence tomography (AS-OCT) with a corneal and epithelial thickness map provides clinicians with a more comprehensive description, useful in both the diagnosis and follow-up of corneal diseases like MECD [8,9]. Indeed, advanced imaging techniques allowed clinicians to perform more reliable differential clinical diagnosis of corneal dystrophies. This task using slit lamps is challenging due to similarities in clinical phenotypes [40,41]. These techniques also can help describe early detections or recurrence characteristics. Epithelial alterations correlate with vision changes [42] and could be analyzed as evidence of pharmacological toxicity [43].

In this study, we present a clinical and genetic analysis of a Spanish family affected by MECD, including AS-OCT images with epithelial maps and IVCM to describe microstructural changes of the cornea.

## 2. Materials and Methods

This case series study was approved by the Institutional Review Board of Centro de Oftalmología Barraquer (IRB/ethics number: 162_MEESMANN2, approval: 7 July 2020). Written informed consent was obtained from all participants before enrolment. The study followed the tenets of the Declaration of Helsinki.

**Figure 1:**
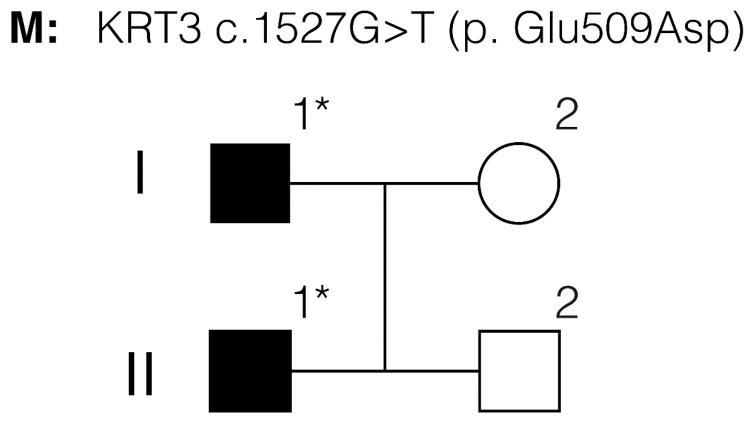
Pedigree of the studied family. Proband is II-1. The asterisks indicate genetically tested individuals.

Three patients were studied: the proband, the proband’s father, and the brother (Figure 1).

The clinical ophthalmologic evaluation included the following: best-corrected visual acuity (BCVA) with ETDRS, direct slit lamp with and without fluorescein and retro illumination, fundoscopy, Schirmer test I (normal value > 10 mm/5 min), non-invasive tear breakup time (NIBUT) (OftalTech, CSO, Florencia, Italia) considering values ≤ 10 s indicative of dry eye disease, and esthesiometry (60 mm maximal measurable sensation) (Cochet-Bonnet, Luneau, France).

Corneal and epithelial thickness maps were evaluated using spectral-domain OCT (Zeiss Cirrus 5000 HD, Zeiss, Dublin, CA, USA).

*The corneal microstructure was* observed by IVCM HRT3 with a Rostock Cornea Module (RCM—Heidelberg Engineering, Heidelberg, Germany). All images were obtained centrally under topical anesthesia with tetracaine (0.1%) and oxybyprocaine chlorhydrate (0.4%) (Alcon Healthcare, S.A., Barcelona, Spain) by the same operator. The best-focused and most representative images were selected, and a mean of 3 images for each patient was considered for the analysis. Epithelial cell density was measured within a region of interest of standardized dimensions (400 × 400 µm). Cells only partially contained in the area analyzed were not counted. The results were expressed in cells per square millimeter (cell/mm^2^). The ImageJ software v1.53e was used to measure the diameter of microcysts.

The genetic analysis was performed by an accredited external laboratory (DBGen Ocular Genomics). Peripheral blood DNA from the patients and available relatives was obtained using the QIAamp DNA Blood Maxi Kit (Qiagen, Hilden, Germany). 

A targeted gene sequence panel was used to study the patients’ DNA using a custom service provider, baits, and developer reagent from Roche, and the resulting fragments were sequenced using an Illumina NovaSeq 6000 SP-2D lane, Illumina, Singapore.

The analysis of the sequences obtained was focused on the coding sequences of the targeted genes (133 genes responsible for dysgenesis and dystrophies of the anterior segment of the eye) and the intronic regions quoted in the panel. Several filtering steps were applied, and the remaining variants were prioritized according to the following criteria: (i) type of variation, (ii) frequency in the control population lower than or equal to 1%, (iii) agreement with the expected inheritance pattern, and (iv) in silico pathogenicity predictors (LRT, MutationTaster, SIFT, PolyPhen2, and CADD). Putative pathogenic variants were then validated using Sanger sequencing. Pathogenicity was considered according to standards and guidelines of the American College of Medical Genetics and Genomics (ACMG) and the Association for Molecular Pathology (AMP).

## 3. Results

The diagnosis of MECD was made based on clinical examination. Three family members of a two-generation family were examined.

The NGS targeted gene panel revealed the proband (Figure 1. patient II-1) and his father (patient I-1) were heterozygous for the novel *KRT3* c.1527G>T p.Glu509Asp genetic variant. This nucleotide substitution had not been previously identified in control populations (Genome Aggregation Database, v.2.1.1). This nucleotide change leads to the amino acid substitution Glu509Asp, located in the highly conserved helix termination motif of the keratin K3 polypeptide. In total, 21 of the 23 in silico tests indicated that this variant presents a deleterious effect. Accordingly, the ACMG guidelines classified this genetic variant as a likely pathogenic. Interestingly, a pathogenic variant has been previously reported affecting the same amino acid codon, *KRT3* c.1525G>A p.E509K [16].

### 3.1. Patient II-1 (Proband)

A 34-year-old man attended our center for photophobia, foreign body sensation, fluctuating vision, and contact lens intolerance. The BCVA was 1.05 (decimal scale) in both eyes. The intraocular pressure (IOP) was within the normal limits (18/17 (OD/OS) mmHg). Esthesiometry showed normal values, Schirmer’s test displayed 30 mm in both eyes, and NiBUTs were within normal values (16.8/17 (OD/OI) s).

**Figure 2:**
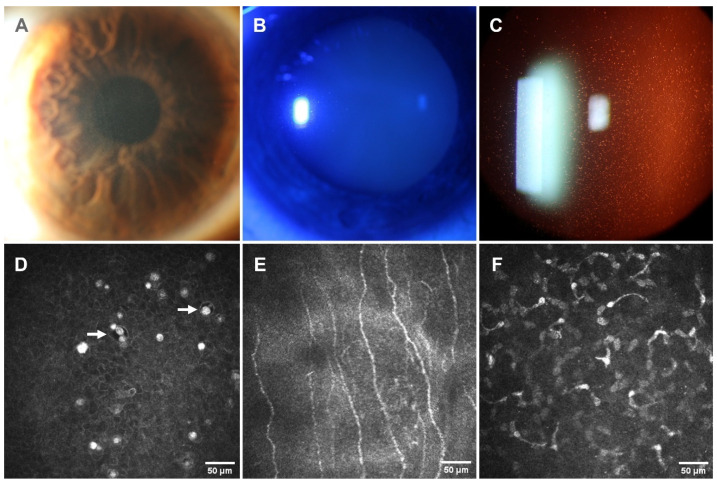
Images of the proband’s right eye (patient II-1). (**A**–**C**): Corneal slit lamp biomicroscopy direct and indirect illumination by dilated pupil displays diffuse and numerous intraepithelial microcysts, and mild geographic and linear subepithelial opacities are observed. (**D**): IVCM image shows intraepithelial microcysts with well-defined edges and hyperreflective materials (arrows) inside and outside of them. (**E**): Tortuous nerves, dendritic cells, and atrophy of Bowman’s layer. (**F**): Active keratocytes present in the deeper stroma.

The slit lamp examination revealed multiple diffuse intraepithelial microcysts in the central interpalpebral area and whitish opacities in both corneas (Figure 2). IVCM images showed intraepithelial microcysts with a small diameter ranging from 12 to 32 µm and a density between 38 and 64 microcysts/mm^2^. Hyperreflective materials, corresponding to cellular debris, were present independently and within the microcysts with densities ranging from 178 to 274 cellular debris/mm^2^. The epithelial cells surrounding them were normal. The presence of tortuous subepithelial nerves, dendritic cells, and atrophy of Bowman’s layer was frequently observed with active keratocytes in the deeper stroma (Figure 2).

**Figure 3:**
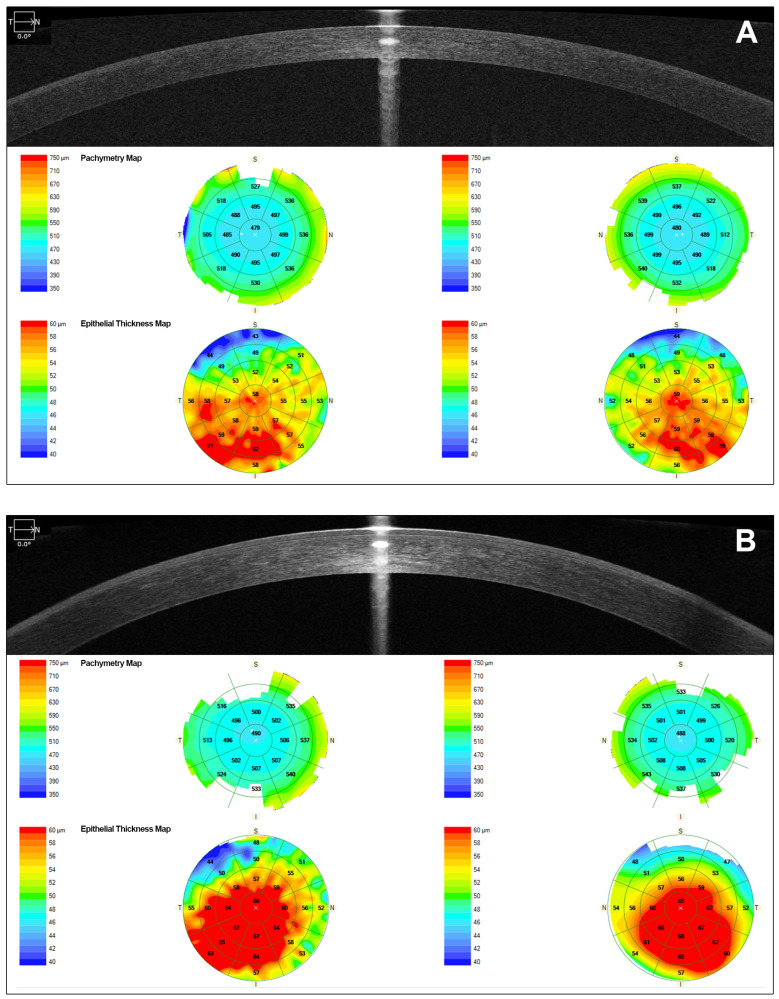
AS-OCT images of the proband’s right eye (patient II-1). (**A**,**B**) Images show diffuse hyperreflectivity in the corneal. (**A**): the epithelial map before MEDC diagnosis, when the patient was wearing contact lenses. (**B**): 8 months after stopping contact lens use, the epithelial map is thickened in the central and inferotemporal areas.

AS-OCT showed diffuse hyperreflectivity in the corneal epithelium and Bowman’s layer. The epithelial map revealed a mean increase in thickness in central (66 OD/65 OI µm) and inferotemporal areas with values ranging from 53 to 68 µm in both eyes (OU). The superior area showed a thinning pattern (ranging from 43 to 51 µm in OU). However, pachymetry displayed a diffuse slight thinning pattern (Figure 3).

### 3.2. Patient I-1

**Figure 4:**
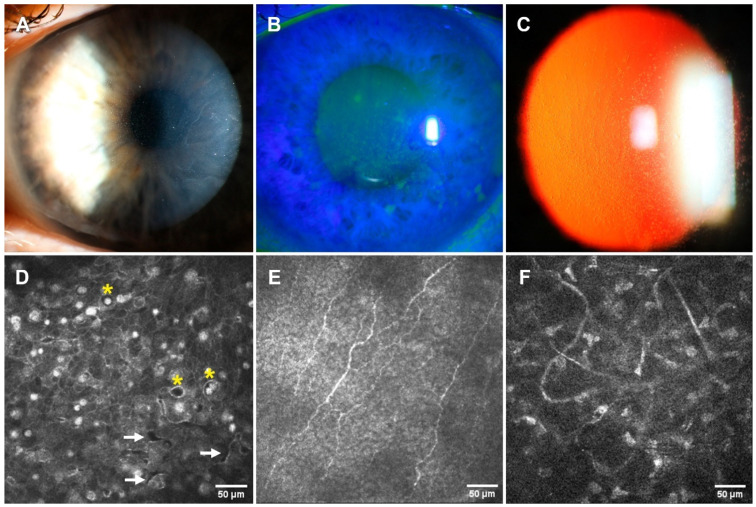
Representative biomicroscopy and IVCM images of the patient’s (I-1) left eye. (**A**–**C**) illustrate small and numerous microcysts, whitish corneal opacities, gray lines, and positive-fluorescein punctate staining. (**D**–**F**): IVCM images. Yellow asterisks indicate microcysts and white arrows indicate epithelial breaks.

This patient was a 62-year-old man who had had mild and fluctuating discomfort since he was 30 years old. Slit lamp examination revealed diffuse central intraepithelial corneal microcysts, whitish corneal opacities, gray lines, and positive-fluorescein punctate staining (Figure 4). Senile cataracts were detected in both eyes and BCVA was 0.65/0.9 (OD/OS) on a decimal scale. Schirmer’s tests were 4/6 mm/5 min (OD/OS) and NiBUT’s were 5.8/9.7 (OD/OS) s. Esthesiometry was within normal values. The patient was previously diagnosed with ocular hypertension and treated with hypotensive medication for 2 years: brimonidine tartrate 2 mg/mL + timolol 5 mg/mL with benzalkonium chloride (*Brimonidina/Timolol Sandoz*; Sandoz Farmaceutica, Madrid, Spain). The IOP was 17/16 mmHg (OD/OI).

IVCM images revealed intraepithelial pleiomorphic cystoid areas with well-defined borders. The microcysts’ diameter ranged from 16.7 to 25.1 µm and there was a density of 83–180 microcysts/mm^2^. The distribution of these lesions was similar to that described in the proband. The density of hyperreflective material was 57–306 cellular debris/mm^2^. Tortuous nerves were observed in the subbasal plexus and the stromal layer, demonstrating extensive Bowman’s layer alteration and keratocyte activation (Figure 4).

**Figure 5:**
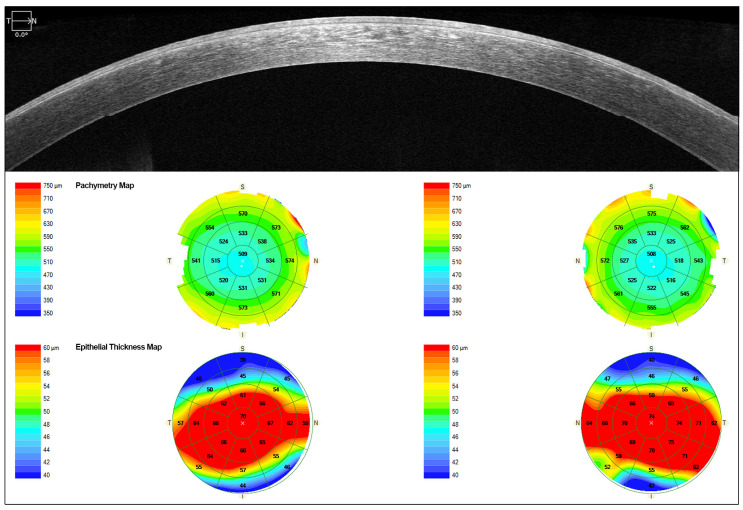
AS-OCT images of the patient’s (I-1) left eye. AS-OCT showed diffuse hyperreflectivity in the corneal epithelium and Bowman’s layer. The epithelial map revealed an increase in thickness at the visual axis and mid periphery, possibly due to chronic inflammation.

AS-OCT scans showed diffuse hyperreflectivity in the epithelium and Bowman’s layer. The epithelial thickness was increased in the interpalpebral area. The mean epithelial central thickness was 70 (OD)/74 (OI) µm and the superior and inferior areas showed a clear thinning with values ranging from 39 to 46 µm in both eyes (Figure 5). The corneal thickness in patient I-1 was slightly thinning (Figure 5).

### 3.3. Patient II-2

The proband’s brother of 27 years of age was asymptomatic and did not present corneal alterations according to the slit lamp examination. The patient declined to perform any genetic analysis.

## 4. Discussion

The findings confirm a new mutation related to the KRT3 gene in two members of a Spanish family with MECD. The patients underwent persistent mild to moderate symptoms with relatively small cysts, Bowman’s layer alterations, subepithelial nerve tortuosity, and signs of stromal inflammation. The epithelial map showed a central inferotemporal thickening pattern with slight thinning in the superior and inferior periphery.

In this sense, a variety of Meesmann genetic mutations have been described worldwide. Since Irvine et al. made the first genetic description associated with MECD in 1997, 6 missense mutations in the *KRT3* gene and 25 missense mutations in the *KRT12* gene have been described as responsible for MECD autosomal dominance. Keratins are structural proteins in the skin epithelium, hair, nails, and cornea. These heteropolymers are assembled from dimers composed of type I and type II intermediate filaments. KRT12 is the type I intermediate filament in the cornea while KRT3 is the type II intermediate filament [16].

In our study, the genetic test revealed a novel likely pathogenic variant in heterozygosity in KRT3 gene c.1527G>T (p.Glu509Asp). Pathogenicity is based on the conservation of the 509-position at the protein level, supportive bioinformatic predictions, and the fact that this variant has not been previously reported in the control population. Finally, two Irish MECD patients presented a pathogenic variant in the same amino acid position [16]. After research in Medline, we did not find any disease other than MECD associated with this mutation or another mutation in KRT3.

To report new MECD mutations could improve disease diagnosis, since the phenotypic expression is variable among different mutations and even among patients from the same family [25]. In addition, knowledge of the mutations reduces the possibility of misdiagnosis, allows a specific gene-based therapy, and helps clinicians provide more specific genetic counseling.

The clinical features in this Spanish family agree with the findings described in the literature [1,11,20,24,29]. As in most previously described cases [7,22,23,27], both patients had mild to moderate symptoms, with the characteristic intraepithelial microcysts [8,9,13,25,31] visualized by biomicroscopy and IVCM. This last technique allows the performing of quantitative measurements. The size of the cysts in the two studied patients was from 12 to 32 µm in diameter, in agreement with Hernandez-Quintela and coworkers’ findings but smaller than described by Patel et al. [3,44,45]. As previously described, we found that the number of microcysts decreased in the epithelial deeper layers closer to the basal layer [9]. We also found the presence of punctate hyperreflective materials and subepithelial nerve tortuosity with extensive Bowman’s layer alterations. These results are in accordance with Nishino and coworkers’ report, suggesting that MECD may affect the epithelium, Bowman’s layer, and stroma, possibly due to chronic inflammation of the epithelium [8]. The older affected patient also showed positive fluorescein corneal staining, probably due to recurrent erosions that match with epithelial breaks shown by IVCM. The presence of punctate corneal erosions may result from intraepithelial microcysts which open spontaneously onto the ocular surface. Nevertheless, we cannot rule out that benzalkonium chloride (BAC), the preservative included in the eye drops for the patient’s IOP control, could have a negative role in the etiology of these lesions. Indeed, BAC, as a quaternary ammonium surfactant, is one of the most used preservatives for eye drops, but it is an irritant [46]. In fact, continuous exposure to BAC can provoke dry eye syndrome by an inflammation induction [47,48,49]. On the other hand, although MECD is considered a stationary condition [37], rupture of the microcysts causing severe pain or subepithelial fibrosis and opacification leading to deterioration of vision in the affected patients can occur, and this worse situation is more frequent in older patients, such as the proband’s father. The most plausible hypothesis is that deterioration in this case was due to different factors. Other imaging methods such as AS-OCT have been used to describe this or other corneal pathologies [3,6,8,9,14,44,45,50]. The corneal epithelial map provided by AS-OCT is a recent tool that could be very useful in this dystrophy, affecting the epithelium and Bowman’s layer. The patients of this study presented a clear increase in epithelial thickness in the inferotemporal cornea (proband) and the interpalpebral area (father’s proband) according to the epithelial map, a similar pattern to the one described by Nishino et al. for the same disease [8]. An inferior and central thickening pattern in the epithelial map has also been reported in patients with epithelial basement membrane dystrophy [41,42], a disease that shares some clinical features with MECD.

In conclusion, we identified a new heterozygous mutation of MECD with autosomal dominant inheritance. A comprehensive characterization of clinical signs using different techniques, especially an epithelial map, could be useful to describe disease progression by quantitative measures. Epithelial map changes provide better understanding of MECD differential epithelial behavior and its progression changes. Larger studies will be necessary for better understanding these specific patterns and clinically evaluating gene-based and other new therapies.

## Figures and Tables

**Table 1:** Reported KRT3 and KRT12 mutations in Meesmann corneal dystrophy.

Gene	Exon	Nucleotide	Amino Acid	References
*KRT3*	1	c.250C>T	p.R84W	Chen JL, et al. (2015) [10]
	7	c.1492G>A	p.G498L	Abad-Morales V, et al. (2021) [15]
	7	c.1493A>T	p.E498V	Szaflik JP, et al. (2008) [14]
	7	c.1508G>C	pR503P	Chen YT, et al. (2005) [18]
	7	c.1525G>A	p.E509K	Irvine AD, et al. (1997) [16]
	7	c.1527G>T	p.E509D	Current report
*KRT12*	1	c.385A>G	p.M129V	Clausen I, et al. (2010) [7]
	1	c.386T>C	p.M129T	Corden LD, et al. (2000) [19]
				Nichini O, et al. (2005) [20]
	1	c.389A>C	p.Q130P	Ogasawara M, et al. (2014) [9]
				Corden LD, et al. (2000) [19]
	1	c.394C>G	p.L132V	Aldave AJ, et al. (2005) [21]
				Nishino T, et al. (2019) [8]
	1	c.395T>C	p.L132P	Liao H, et al. (2011) [22]
				Hassan H, et al. (2013) [23]
	1	c.395T>A	p.L132H	Wang LJ, et al. (2007) [12]
	1	c.403A>G	p.R135G	Nishida K, et al. (1997) [17]
	1	c.404G>T	p.R135I	Nishida K, et al. (1997) [17]
	1	c.404G>C	p.R135T	Irvine AD, et al. (1997) [16]
				Corden LD, et al. (2000) [19]
				Ehlers N, et al. (2008) [25]
	1	c.405A>C	p.R135S	Yoon MK, et al. (2004) [26]
	1	c.409G>C	p.A137P	Takahashi K, et al. (2002) [29]
	1	c.419T>G	p.L140R	Nishida K, et al. (1997) [17]
	1	c.419T>A	p.L140Q	Ogasawara M, et al. (2014) [9]
	1	C.423T>G	p.N133K	Irvine AD, et al. (2002) [24]
	1	c.427G>C	p.V143L	Irvine AD, et al. (1997) [16]
	1	c.427G>T	p.V143L	Nielsen K, et al. (2008) [6]
	6	1222ins27bp	p.400ins9	Yoon MK, et al. (2004) [26]
	6	c.1276A>G	p.I426V	Coleman CM, et al. (1999) [11]
	6	c.1277T>G	p.I426S	Nichini O, et al. (2005) [20]
	6	c.1285T>G	p.Y429D	Nishida K, et al. (1997) [17]
	6	c.1286A>G	p.Y429C	Chen YT, et al. (2005) [18]
	6	c.1288_1293delins6bp	p.R430_R431delins-SP	Chen JL, et al. (2015) [10]
	6	c.1289G>C	p.R430P	Sullivan LS, et al. (2007) [27]
	6	c.1298T>G	p.L433R	Seto T, et al. (2008) [28]
	6	c.1273G>A	p.G425L	Dong PN, et al. (2020) [13]

## Data Availability

The original contributions presented in this study are included in the article. Further inquiries can be directed to the corresponding author.
